# 229. Molecular and Clinical Epidemiology of Carbapenemase-Producing *E. coli* Isolates from Different Global Regions (CRACKLE-2)

**DOI:** 10.1093/ofid/ofac492.307

**Published:** 2022-12-15

**Authors:** Angelique E Boutzoukas, Angelique E Boutzoukas, Lauren Komarow, Liang Chen, Keri R Baum, Vance G Fowler, Carol Hill, Cesar A Arias, Blake M Hanson, David Paterson, Lizhao Ge, Karen M Ordonez, Soraya Salcedo Mendoza Salcedo, Souha S Kanj, Robert Bonomo, David van Duin

**Affiliations:** Duke University School of Medicine, Durham, North Carolina; Duke University School of Medicine, Durham, North Carolina; George Washington University, Rockville, Maryland; HMH-CDI, Nutley, New Jersey; Duke Clinical Research Institute, Durham, North Carolina; Duke University Medical Center, Durham, North Carolina; Duke Clinical Research Institute, Durham, North Carolina; Houston Methodist Hospital and Weill Cornell Medical College, Houston, TX; University of Texas Health Science Center, Houston, Texas; The University of Queensland, Brisbane, Queensland, Australia; George Washington University, Rockville, Maryland; Hospital Universitario San Jorge de Pereira, Pereira, Risaralda, Colombia; Clínica General del Norte, Barranquilla, Atlantico, Colombia; American University of Beirut Medical Center, Beirut, Beyrouth, Lebanon; Case Western University, Cleveland, Ohio; University of North Carolina at Chapel Hill, Chapel Hill, North Carolina

## Abstract

**Background:**

Carbapenemase-producing (CP) *Escherichia coli* (CP*Ec*) are a global public health threat. We describe the epidemiology and outcomes of patients with CP*Ec* isolates obtained in CRACKLE 2, a prospective cohort study of hospitalized patients with positive cultures for CP Enterobacteriaceae.

**Methods:**

In CRACKLE-2, patients with CP*Ec* were enrolled from 26 hospitals in 6 countries (ClinicalTrials.gov NCT03646227). Clinical data were collected, and bacterial isolates underwent whole genome sequencing (WGS). Here, we included unique patients with CP*Ec* by WGS (n=114). The primary outcome was desirability of outcome ranking (DOOR) at 30 days after index culture. Chi squared tests with alpha = 0.05 were used to evaluate differences in culture source and outcomes between metallo-beta-lactamase (MBL) and non-MBL isolates.

**Results:**

Of 114 CP*Ec* isolates, 57 (50%) represented infection (**Table 1**). Isolates primarily arose from urine (34%) and blood (21%). Compared to non-MBL isolates, isolates containing MBL were more often from urine (41% vs 29%) and less frequently from blood (6% vs 32%); p=0.02. We observed strong regional variations in CP (Figure 1) and MBL (p < 0.0001). Sequence type (ST) 167 was more common among MBL than non-MBL isolates (31% vs 2%, p< 0.0001); non-MBL isolates were more often ST410 and ST131 (17% and 20%). Extended-spectrum beta-lactamases (ESBL) were present in 52% of isolates; most commonly, CTX-M-15 in both MBL (33%) and non-MBL isolates (34%). Phylogenetic analysis of the isolates and corresponding region, bacterial characteristics, and DOOR outcomes are in **Figure 1**. Death at 30 days occurred in 18 (16%) of patients, more commonly among non-MBL than MBL CP*Ec* (23% vs 6%; p=0.01). The probability of a better DOOR outcome for a randomly selected MBL was 58% [95% CI: 48.2, 67.4], indicating no significant difference between the groups.

**Figure:**
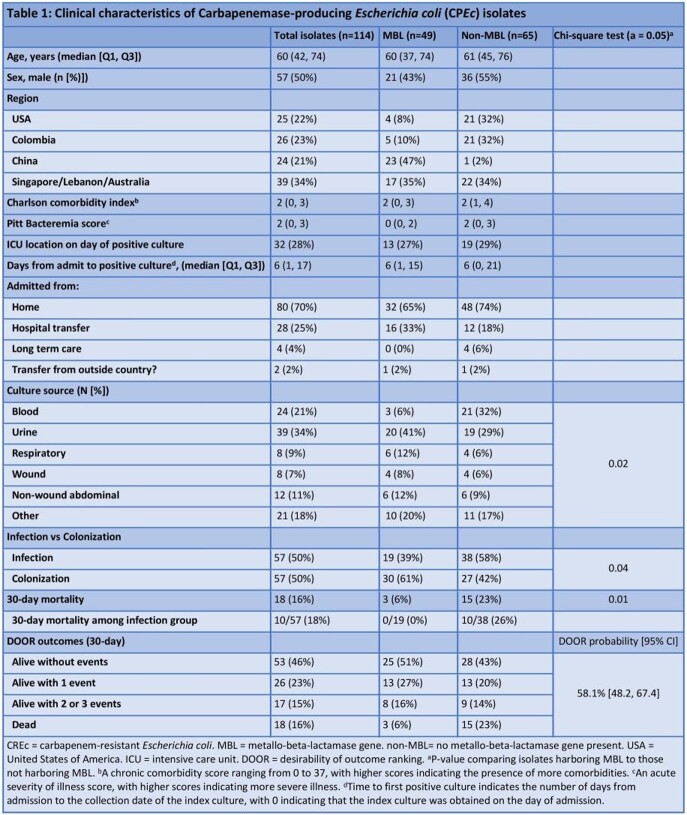

**Figure 1:**
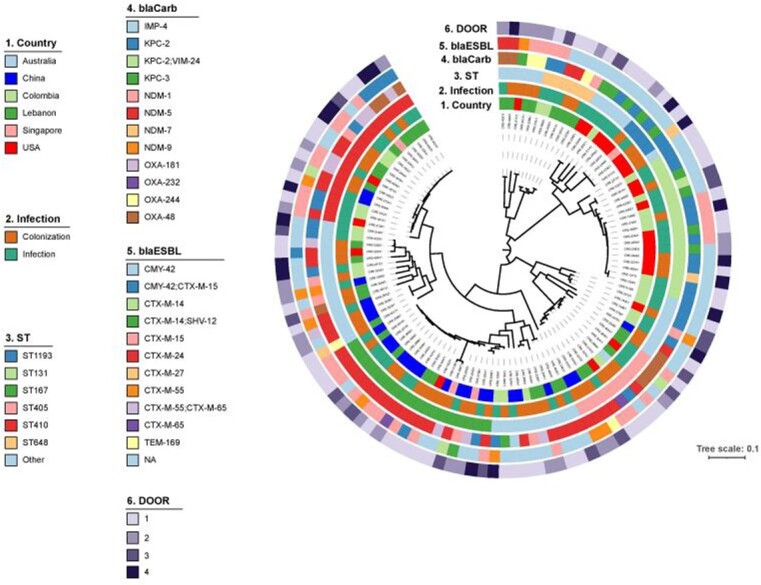
Phylogenetic population structures of Carbapenemase-producing Escherichia coli (CPEc) isolates

Legend: Infection = categorization as infection or colonization. ST = sequence type. BlaCarb = Carbapenemase gene. BlaESBL = acquired ESBL enzymes. DOOR = desirability of outcome ranking. DOOR rankings: 1 = Alive without events; DOOR 2 = Alive with 1 event; DOOR 3 = Alive with 2 or 3 events; DOOR 4 = dead.

**Conclusion:**

Emergence of carbapenem resistance with important geographic variations was observed in *E coli* including among high-risk clones such as ST131. Mortality was higher among non-MBL isolates, which were more frequently from blood, but these findings may be confounded by region.

**Disclosures:**

**Vance G. Fowler, Jr, MD, MHS**, Affinergy: Grant/Research Support|Affinergy: Honoraria|Affinium: Honoraria|Amphliphi Biosciences: Honoraria|ArcBio: Stocks/Bonds|Basilea: Grant/Research Support|Basilea: Honoraria|Bayer: Honoraria|C3J: Honoraria|Cerexa/Forest/Actavis/Allergan: Grant/Research Support|Contrafect: Grant/Research Support|Contrafect: Honoraria|Cubist/Merck: Grant/Research Support|Debiopharm: Grant/Research Support|Deep Blue: Grant/Research Support|Destiny: Honoraria|Genentech: Grant/Research Support|Genentech: Honoraria|Integrated Biotherapeutics: Honoraria|Janssen: Grant/Research Support|Janssen: Honoraria|Karius: Grant/Research Support|Medicines Co.: Honoraria|MedImmune: Grant/Research Support|MedImmune: Honoraria|NIH: Grant/Research Support|Novartis: Grant/Research Support|Novartis: Honoraria|Pfizer: Grant/Research Support|Regeneron: Grant/Research Support|Regeneron: Honoraria|Sepsis diagnostics: Sepsis diagnostics patent pending|UpToDate: Royalties|Valanbio: Stocks/Bonds **Cesar A. Arias, MD, PhD**, Entasis Phramceuticals: Grant/Research Support|MeMed Diagnostics: Grant/Research Support|Merck: Grant/Research Support **David Paterson, MBBS**, Accelerate: Honoraria|bioMerieux: Honoraria|Entasis: Advisor/Consultant|Janssen-Cilag: Grant/Research Support|MSD: Advisor/Consultant|MSD: Grant/Research Support|MSD: Honoraria|Pfizer: Grant/Research Support|Pfizer: Honoraria|PPD: Grant/Research Support|Shionogi: Grant/Research Support|VenatoRx: Advisor/Consultant **Karen M. Ordonez, MD**, AstraZeneca: Expert Testimony|Biomerieux: Expert Testimony|Farma de Colombia: Expert Testimony|MSD: Expert Testimony|Pfizer: Expert Testimony **Souha S. Kanj, Pr**, MSD, Pfizer, Gilead, Menarini, Astellas: Advisor/Consultant|MSD, Pfizer, Gilead, Menarini, Astellas: Honoraria **Robert Bonomo, MD**, Cystic Fibrosis Foundation: Grant/Research Support|Merck: Grant/Research Support|NIH: Grant/Research Support|VA: Grant/Research Support|VenatoRx: Grant/Research Support|Wockhardt: Grant/Research Support **David van Duin, MD, PhD**, Achaogen: Advisor/Consultant|Allergan: Advisor/Consultant|Astellas: Advisor/Consultant|MedImmune: Advisor/Consultant|Melinta: Advisor/Consultant|Merck: Advisor/Consultant|Merck: Grant/Research Support|NeuMedicine: Advisor/Consultant|Pfizer: Advisor/Consultant|Qpex: Advisor/Consultant|Roche: Advisor/Consultant|Sanofi-Pasteur: Advisor/Consultant|Shionogi: Advisor/Consultant|Shionogi: Grant/Research Support|T2 Biosystems: Advisor/Consultant|Tetraphase: Advisor/Consultant.

